# Evaluating head CT referral quality and appropriateness in an Italian emergency department: a monocentric retrospective study

**DOI:** 10.1186/s13244-025-02042-1

**Published:** 2025-08-26

**Authors:** Marco Parillo, Milena Calabrese, Anna Maria Careddu, Antonio Alessandro Pallottino, Carlo Cosimo Quattrocchi

**Affiliations:** 1 Radiology, Multizonal Unit of Rovereto and Arco, APSS Provincia Autonoma Di Trento, Trento, Italy; 2 Radiology, Santa Chiara Hospital, APSS Provincia Autonoma Di Trento, Trento, Italy; 3 Centre for Medical Sciences—CISMed, University of Trento, Trento, Italy

**Keywords:** Practice Guideline, Referral, Tomography (X Ray Computed), Emergency Departments, Brain

## Abstract

**Objectives:**

To analyze the quality and appropriateness of head CT referrals from the emergency department (ED) of a single hospital in Italy.

**Materials and methods:**

A quality care study was designed to retrospectively identify consecutive head CT referrals generated from the ED of a tertiary hospital between January 1 and April 30, 2022. Referral quality was assessed using the Reason for Exam Imaging Reporting and Data System (RI-RADS), while referral appropriateness was evaluated according to the American College of Radiology (ACR) criteria.

**Results:**

We included 2908 imaging requests, of which 620 (21%) were adequate (RI-RADS A or B) and 2288 (79%) were inadequate (RI-RADS C or D) in terms of quality. In 410 cases, it was not possible to evaluate the appropriateness of the requests according to the ACR guidelines due to the lack of clinical data. Among the 2498 evaluable requests, 25% were classified as usually not appropriate. Of the requests with RI-RADS A or B, 84% were appropriate. Conversely, among the evaluable requests with RI-RADS C or D, the percentage of appropriate requests dropped to 70%. Of all patients with inappropriate requests, 98% did not suffer from acute cerebral diseases according to imaging, with headache and syncope being the primary clinical indications. Analysis of positivity rates revealed a significant difference between appropriate and inappropriate CT scans (11% vs 1%; *p*-value < 0.001).

**Conclusion:**

The recent increase in head CT scan requests in the ED is not completely justified and could be mitigated by improving the quality and appropriateness of referrals.

**Critical relevance statement:**

Excessive head CT requests in the ED cause needless radiation, pollution, and costs. Integrating guidelines and prospective justification with clear documentation in patient records, along with improved staff training and a no-blame culture, are key to reducing unnecessary imaging.

**Key Points:**

- RI-RADS scores the quality, while the ACR criteria assess the appropriateness of imaging referrals.
- Most ED head CT requests lacked quality (especially lacking a specific diagnostic question) and were often clinically inappropriate.
- Appropriate head CT indication strongly predicted finding acute cerebral pathology on imaging.

**Graphical Abstract:**

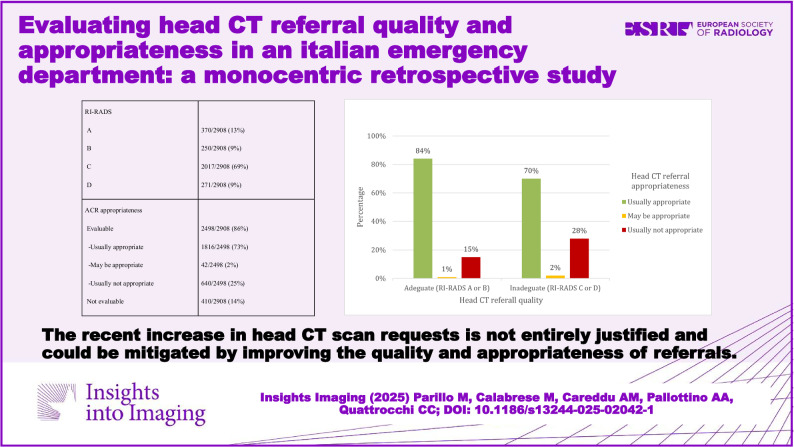

## Introduction

Computed tomography (CT) is a key diagnostic tool in the emergency department (ED) for triage of critical patients, helping to establish or rule out acute diseases. Over recent years, the use of CT has significantly increased, with a 30% rise since 2007 [1]. In our radiology department, the number of head CT referrals has increased 2.4-fold between 2008 and 2023. This increase has led to higher healthcare costs and increased exposure to ionizing radiation, which must be justified by the diagnostic and therapeutic benefits of CT scanning [2, 3]. Maintaining current trends in CT usage could lead to CT-related cancers representing as much as 5% of all newly diagnosed cancers each year [1]. Beyond the direct health concerns, it is crucial to consider the environmental consequences of CT scans. The electrical power consumed during a CT examination and the resulting ecological footprint from greenhouse gas emissions, such as CO_2_, contribute negatively to the environment [4]. The growing use of CT can be attributed to both the technological advancements in imaging and the practice of defensive medicine, where medical actions are often driven by legal concerns rather than clinical necessity [5]. While most studies in the literature focus on head CTs performed in trauma cases, which are the most common, fewer studies analyze all head CT requests in the ED. Some studies analyzing all CT scans performed in EDs have found that head CT scans constitute the majority of those performed, encompassing both traumatic and non-traumatic clinical conditions. Among these head CT scans, up to 75% are reported as negative [6–9].

Diagnostic imaging requests serve as the main channel of communication between the referring physician and the radiologist. On one hand, imaging requests must be clinically appropriate to ensure proper justification. Conversely, thoroughness in these requests is essential, as the omission of key clinical information can significantly increase the risk of diagnostic errors, potentially leading to severe patient outcomes [10]. To support referring clinicians in selecting optimal imaging modalities, the American College of Radiology (ACR) has established evidence-driven appropriateness guidelines. These standards are subject to yearly evaluation and revision by panels of specialists in diagnostic and interventional radiology, which comprise leading experts from both radiological and other relevant medical fields [11, 12]. Based on the ACR appropriateness criteria, the standalone European Society of Radiology (ESR) iGuide portal grants access to the ESR’s imaging referral guidelines [13, 14]. To enhance the quality and consistency of diagnostic imaging referrals, the Reason for Exam Imaging Reporting and Data System (RI-RADS) has been introduced. This system provides a standardized framework for the clinical data included in radiology requests, enabling radiologists to evaluate the sufficiency of the supplied information. Their assessment, incorporated into the radiology report, utilizes a five-tier scale, examining three primary components: “impression”, “clinical information”, and “diagnostic question” [15, 16]. Providing both corrective feedback for inadequate requests and positive reinforcement for exemplary ones may encourage physicians to refine their practice, resulting in a higher standard of submitted imaging referrals [17].

The current study intends to analyze the quality (via RI-RADS) and appropriateness (following ACR guidelines) of head CT referrals from the ED of a single hospital in Italy.

## Methods

### Study design and patient selection

The ethics committee approved this retrospective study of observational data (approval number: 2024-087ESA), in compliance with the 2013 Declaration of Helsinki. Because the analysis relied solely on previously gathered and anonymized data from patient records, informed consent was waived.

One radiologist (4 years of experience) retrospectively reviewed all consecutive head CT radiology requests generated from the ED of a tertiary hospital within the Italian public healthcare system between January 1 and April 30, 2022.

For each patient, data collected from the electronic health record system included gender, age, the original head CT requests, the initial head CT report, and findings reported from any follow-up head CT or magnetic resonance imaging (MRI) studies (including angiography) within 30 days.

Head imaging reporting acute brain diseases was classified as positive. Head imaging reported as negative, as well as those showing non-acute findings such as brain atrophy, non-recent ischemic sequelae, vasculopathy, or gliosis, were all classified as negative.

### Quality of head CT referrals

Head CT referral quality was assessed by a radiologist (4 years of experience) using the RI-RADS scale (A–D), with requests categorized as adequate (A and B) or inadequate (C and D). For this evaluation, we evaluated only the information provided in the imaging requests; no additional data from alternative sources were taken into account.

Briefly, the RI-RADS grade is assigned using three key categories: (1) the “impression” (e.g., preliminary interpretation or differential diagnosis), (2) the “clinical information” (e.g., signs, symptoms, and pertinent past medical history), and (3) the “diagnostic question” (e.g., confirming or excluding a specific diagnosis). The RI-RADS A grade reflects full inclusion of all three key informational components. RI-RADS B indicates that all three components are included, but with some gaps in clinical information. RI-RADS C signifies that only two key components are represented. RI-RADS D denotes that only a single, or no, key informational component is provided [15]. This study employed the early RI-RADS version, combining category X (absence of key categories) with D, as all such requests exhibited inadequate diagnostic quality. See Table 1 for an indicative example of RI-RADS application.

**Table 1:** Example of RI-RADS application

Grade	Description	Key categories* included in the request	Example of RI-RADS A for a head CT referral
RI-RADS A	Adequate	All key categories included	Impression: left hemiparesis syndrome. Clinical information: 70-year-old female with left-sided weakness and contralateral paresthesia (onset 1 h prior); no chronic medications or surgical history; vital signs, including blood pressure and heart rate, within normal limits; positive left Mingazzini test and dysmetria on index-nose test; and normal chest examination. Diagnostic question: rule out stroke.
RI-RADS B	Barely adequate	All key categories are included, but some clinical information is missing	
RI-RADS C	Considerably limited	Two key categories included	
RI-RADS D	Deficient	One or no key category included	

* RI-RADS key categories

1. Impression: working or differential diagnosis

2. Clinical information: signs and symptoms, the time course of the current event, localization of clinical manifestations, relevant antecedent medical and surgical procedures, pertinent laboratory data, and, if available, previous imaging records

3. Diagnostic question: confirm or rule out a condition, determine disease severity, guide surgical planning, monitor disease progression, or assess treatment efficacy

### Appropriateness of head CT referrals

Head CT referral appropriateness was evaluated by a radiologist (4 years of experience) according to the criteria of the ACR [18–28]. Table 2 summarizes the main clinical indications and corresponding appropriateness levels for requesting unenhanced head CT.

**Table 2:** Summary of the main clinical indications and corresponding appropriateness for requesting unenhanced head CT, according to the ACR criteria

Clinical indication	Clinical picture	Appropriateness
Traumatic brain injury (adults or children aged 16 years and older)	Mild head trauma (GSC 13–15)* with: • Loss of consciousness or post-traumatic amnesia, with: headache, vomiting, age > 60 years, drug or alcohol intoxication, short-term memory loss, physical evidence of trauma above the clavicle, post-traumatic seizure, GCS < 15, focal neurological deficit, and/or coagulopathy; • Without loss of consciousness or post-traumatic amnesia, with: focal neurological deficit, vomiting, severe headache, age ≥ 65 years, physical signs of basicranial fracture, GCS < 15, coagulopathy, and/or high-energy trauma	Usually appropriate
	Moderate (GCS 9–12), severe (GCS 3–8), or penetrating head trauma	Usually appropriate
Traumatic brain injury (children < 16 years)	Mild (GCS = 15) and very low risk for clinically important brain injury°	Usually not appropriate
	Mild (GCS = 15) and intermediate risk for clinically important brain injury°	May be appropriate
	Mild (GCS = 14) and high risk for clinically important brain injury°	Usually appropriate
	Moderate or severe (GCS ≤ 13) head trauma	Usually appropriate
Stroke or stroke-related conditions	Transient ischemic attack	Usually appropriate
	Focal neurologic deficit	Usually appropriate
	Suspected venous sinus thrombosis	Usually appropriate
	Suspected cervical vascular dissection or injury	Usually appropriate
Headache	Severe headache (“the worst headache of one’s life”) or headaches with: sudden onset (or “thunderclap”), signs of intracranial hypertension or hypotension, new pattern or onset during pregnancy or the peripartum period, increased frequency or severity, fever or neurological deficits, history of cancer or immunosuppression, onset after age 50, and/or post-traumatic onset (within the last 7 days)	Usually appropriate
	Classic migraine or tension-type primary headache	Usually not appropriate
	Trigeminal neuralgia	Usually not appropriate
Dizziness	Brief episodic vertigo triggered by head movements	Usually not appropriate
	Acute persistent vertigo with normal neurological examination and a HINTS test consistent with peripheral vertigo	Usually not appropriate
	Acute persistent vertigo with abnormal neurological examination and a HINTS test consistent with central vertigo	May be appropriate
	Chronic recurrent vertigo with unilateral hearing loss or tinnitus	Usually not appropriate
	Chronic recurrent vertigo with brainstem neurological deficits	May be appropriate
Syncope or pre-syncope	Absence of traumatic brain injury	Usually not appropriate

*GSC* Glasgow Coma Scale, *HINT* head impulse, *Nystagmus* test of skew

* In the case of mild TBI, the ACR defers to hospital protocols or, where those are absent, to the 2008 Clinical Policy of the American College of Emergency Physicians (ACEP)

° In the case of mild TBI, the ACR defers to the Pediatric Emergency Care Network (PECARN) criteria to classify the risk of clinically important brain injury

### Statistical analysis

Statistical analysis was conducted using the chi-square test with Yates' correction. The sensitivity, specificity, positive predictive value (PPV), and negative predictive value (NPV) of appropriate imaging in identifying acute brain diseases were determined for the most clinical indications in the study population. Additionally, the relative risk (RR) of undergoing further diagnostic procedures (CT angiography and MRI) according to examination appropriateness was assessed. Statistical significance was set at a *p*-value threshold of 0.05.

## Results

A total of 2908 head CT requests were included in the study. The most frequent clinical indications among the examined requests were trauma (38%) and headache (16%). Most head CT scans were reported as negative for urgent brain findings (92%). Among the CT scans reported as positive, subarachnoid hemorrhage was the most frequently described finding. Furthermore, the majority of patients did not undergo CT angiography or MRI within 30 days of their ED admission. Table 3 summarizes the data included in the study.

**Table 3:** Distribution of data included in the study

Variables	Count
Total unenhanced head CT requested	2908
Patient demographics	
Male/female	1423/1485
Mean age in years ± standard deviation	60 ± 23
Age < 16 years	90/2908 (3%)
Clinical indication	
Trauma	1103/2908 (38%)
Headache	466/2908 (16%)
New-onset neurological deficit	308/2908 (10%)
Dizziness	238/2908 (8%)
Syncope	163/2908 (6%)
Hemisyndrome	134/2908 (5%)
Delirium	79/2908 (3%)
Seizure	75/2908 (3%)
Non-focal neurological deficit	65/2908 (2%)
Stupor	39/2908 (1%)
Miscellaneous	102/2908 (3%)
Unspecified	136/2908 (5%)
CT findings on the first scan	
Negative	2680/2908 (92%)
Positive	228/2908 (8%)
Subarachnoid hemorrhage	64
Infarction	47
Intraparenchymal hemorrhage	43
Subdural hematoma	41
Skull fracture	37
Neoplasm	20
Intraventricular hemorrhage	14
Microbleed	13
Thrombus	10
Hydrocephalus	5
Herniation	3
CT angiography	
Performed	260/2908 (9%)
Not performed	2648/2908 (91%)
MRI	
Performed	164/2908 (6%)
Not performed	2744/2908 (94%)

*CT* computed tomography, *MRI* magnetic resonance imaging, *RI-RADS* reason for exam imaging reporting and data system, *ACR* American College of Radiology

### Quality and appropriateness of head CT referrals

Out of 2908 head CT scan requests, 620 (21%) were adequate (RI-RADS A or B), and 2288 (79%) were inadequate (RI-RADS C or D) in terms of quality. Notably, the majority of the requests (76%) lacked a diagnostic question. Common requests classified as RI-RADS C included: “head CT for traumatic brain injury on novel oral anticoagulants”; “head CT for headache not responding to analgesics”.

In 410 (14%) requests, it was not possible to evaluate the appropriateness of the requests according to the ACR guidelines due to the lack of clinical data in the requests themselves. Of these, 43 were classified as RI-RADS A or B and 367 as RI-RADS C or D. Among the 2498 evaluable requests, the majority (73%) were classified as usually appropriate (Table 4).

**Table 4:** RI-RADS quality grade and ACR appropriateness of head CT referrals assigned in the study

RI-RADS grade	
A	370/2908 (13%)
B	250/2908 (9%)
C	2017/2908 (69%)
D	271/2908 (9%)
RI-RADS key categories	
Impression (presence/absence)	2767/141
Clinical information (presence/absence)	2660/248
Diagnostic question (presence/absence)	698/2210
ACR appropriateness	
Evaluable	2498/2908 (86%)
Usually appropriate	1816/2498 (73%)
May be appropriate	42/2498 (2%)
Usually not appropriate	640/2498 (25%)
Not evaluable	410/2908 (14%)

**Fig. 1:**
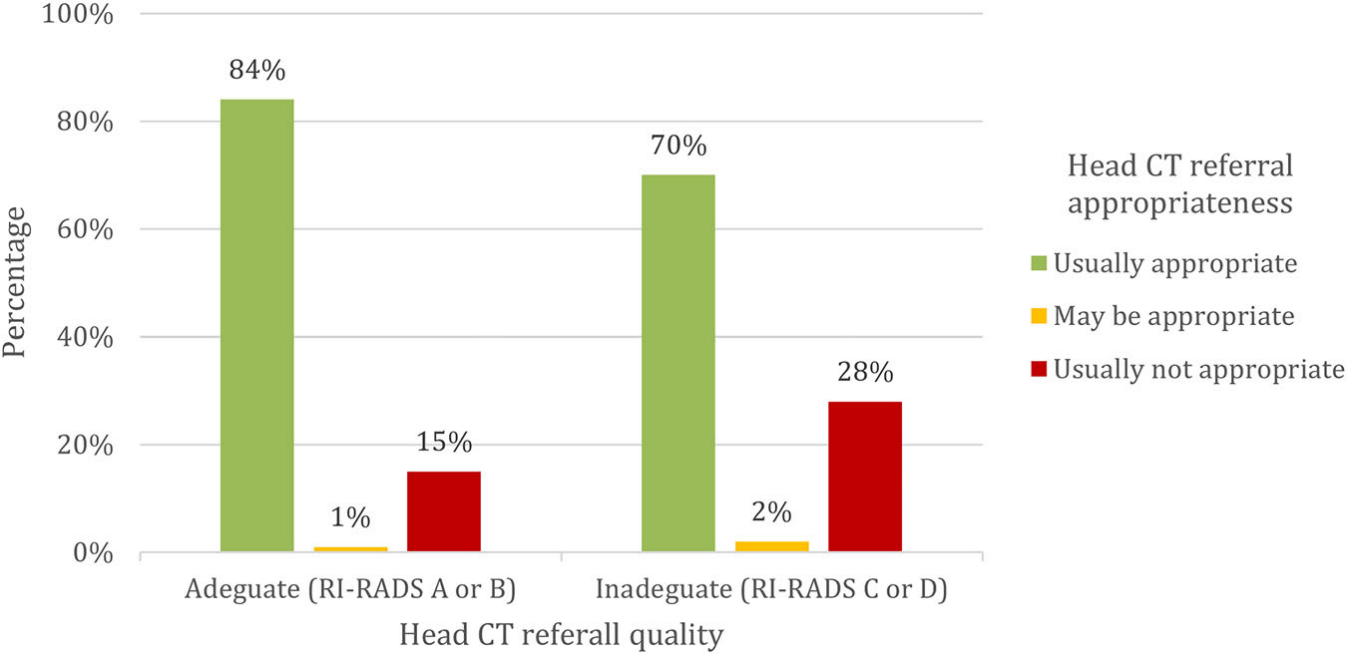
Column chart showing the percentage of appropriateness of head CT requests according to ACR guidelines, grouped by the quality of the requests according to RI-RADS

Of the evaluable requests with RI-RADS A or B, 84% were appropriate according to ACR criteria. Conversely, among the evaluable requests with RI-RADS C or D, the percentage of appropriate requests dropped to 70% (Fig. 1).

### Correlation between quality and appropriateness of head CT referrals and outcomes

Among inappropriate requests (according to ACR criteria) for head CT classified as RI-RADS A or B, 96% were negative. However, 4 patients had positive imaging findings, including: 1 tumor, 1 subgaleal hematoma, 1 arteriovenous malformation, and 1 intraparenchymal hemorrhage (diagnosed on a follow-up CT scan). The main clinical indications among the inappropriate requests were headache (26%) and trauma (25%).

Of patients with inappropriate requests (according to ACR criteria) for head CT classified as RI-RADS C or D, 99% were negative. However, 8 patients had positive imaging findings, including: 3 tumors and 1 subarachnoid hemorrhage on the initial CT scan, 1 aneurysm and 2 carotid stenoses on CT angiography, and 1 ischemia on MRI. The main clinical indications among the inappropriate requests were syncope (22%) and headache (22%).

Of all patients with inappropriate requests for head CT, 98% were negative, with headache (23%) and syncope (21%) being the primary clinical indications. Conversely, among the patients with appropriate requests, 16% were positive, yielding 198 positive findings on the initial CT scan, 29 on the 24-h follow-up CT, 31 on CT angiography, and 24 on MRI. The main clinical indications for appropriate requests were trauma (47%) and stroke (23%).

Analysis of positivity rates revealed a significant difference between appropriate and inappropriate CT scans (11% vs 1%; *p*-value < 0.001), highlighting that appropriate head CT scans had a significantly higher rate of reporting acute cerebral diseases compared to inappropriate scans.

Appropriate CT requests were significantly more likely to require further diagnostic investigation with CT angiography (RR 1.4, 95% confidence interval: 1.3–1.4; *p*-value < 0.001) and MRI (RR 1.3, 95% confidence interval: 1.2–1.3; *p*-value < 0.001) compared to inappropriate requests.

**Fig. 2:**
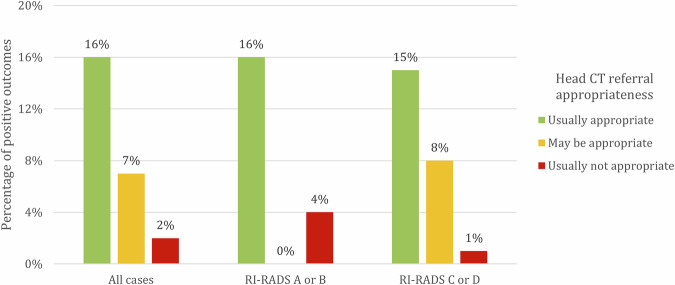
Column chart showing the percentage of positive outcomes (patients with acute cerebral radiological findings) in relation to the appropriateness criteria of the ACR guidelines, across all cases and grouped by the quality of the requests according to the RI-RADS

When analyzing the positivity rate according to clinical indication (Table 4), for head trauma, 85 appropriate CT scans showed positive findings (Fig. 2), whereas no inappropriate CT scan was positive. This resulted in a PPV of 1 and an NPV of 0.1 (*p*-value < 0.01). In cases of headache, 11 appropriate CT scans were positive, and one inappropriate scan was positive. When evaluating new-onset focal neurological deficits, 31 appropriate CT scans yielded positive results, while only one inappropriate CT scan showed positive findings. For patients with dizziness, a single positive CT scan was observed in both the appropriate and inappropriate request groups.

## Discussion

This study shows that the majority of unenhanced head CT requests in the ED were inadequate in terms of quality (79%), especially when a specific diagnostic question is missing, with a high rate of inappropriateness in terms of clinical indication (25% considering only the referrals that could be correctly evaluated). The risk of inappropriate head CT scans could be mitigated by improving the quality of requests, as suggested by the lower number of appropriate requests scored as RI-RADS C or D. This can be attributed to the difficulty emergency physicians face in applying guidelines without a clear diagnostic query. Furthermore, defensive medicine, which is widespread but especially prevalent in emergency settings, plays a significant role, with CTs often requested under the false assumption of improved legal standing.

Headache and syncope, accounting for roughly half of all inappropriate requests, were identified as the leading clinical indications for inappropriateness. This highlights the necessity for enhanced training and education on appropriate CT utilization, particularly in these clinical situations.

Moreover, appropriate indication was associated with a higher likelihood of identifying cerebral findings and, considering the most frequent clinical indications, showed a high predictive value for the actual presence of an acute cerebral pathology on radiological examination. This reinforces the notion that the recent increase in head CT scan requests in the ED is not justified. Inappropriate CT procedures represent an unnecessary expense in economic, environmental, and unjustified radiation exposure terms. In particular, the issue of radiation exposure has received considerable attention nowadays, exemplified by the 2013/59/EURATOM directive outlining basic safety measures for protection from ionizing radiation hazards [29], which Italy implemented via Legislative Decree 101/2020 [30]. According to Article 159 of the decree, the radiologist is the professional who bears primary responsibility for evaluating the appropriateness of radiological examinations, including CT scans. Ideally, the process of requesting a radiological examination begins with a motivated request from the referring physician (prescribing physician), which highlights the clinical necessity of the examination. The radiologist then evaluates this request, taking into account the clinical information provided, the suitability of the requested examination to answer the clinical question, and the possibility of using alternative imaging modalities that involve lower radiation exposure. The radiographer also plays an important role in verifying the consistency between the clinical question and the prescription and can report cases that do not generally appear justified to the radiologist for further evaluation. Although this workflow is desirable, it is often difficult to apply in daily clinical practice, especially in the emergency setting, when refusing to perform a radiological examination based on the aforementioned dictates could, although rarely, lead to significant clinical consequences for the patient. Furthermore, the daily heavy workload makes a detailed evaluation of all radiological requests difficult for a single radiologist, and radiographers cannot be sufficiently trained to assess appropriateness in all cases.

Previous studies have partially examined this topic. Ferorelli et al examined 100 head CT requests originating from the ED. Appropriateness was assessed retrospectively and blindly by a panel of expert radiologists, neurologists, and anesthesiologists. The results indicated that 32% of the CT examinations were deemed inappropriate, with 6% of these attributed to defensive medical practices [8]. A retrospective analysis by Nishtar et al examined all head CT scans conducted at an ED within a Pakistani hub center from November 1, 2017, to January 31, 2018. The analysis of 3893 scans revealed a 33.7% positivity rate, with the majority of positive findings related to major trauma or neurological deficits in elderly patients. The study argued for CT overuse, resulting in increased costs and radiation exposure, but its conclusions were drawn solely from positivity rates, without evaluating appropriateness [7]. The Ethiopian study by Demeke and Mekonnen aimed to identify the extent of inappropriate cranial CTs by evaluating all CTs performed during a given period in the radiology department of a specialized hospital, including both emergency/urgent and scheduled CTs. It considered only requests with a complete or almost complete set of the required information (> 80% of the information). The final sample comprised 443 CTs. The appropriateness was evaluated according to the ACR guidelines: 51.9% fell into the “usually appropriate” category, 15.1% were considered “possibly appropriate.”, and 11.7% were deemed inappropriate. In the remaining 21.3%, the requests did not fit into any of the ACR criteria categories. Headaches (38.5%), seizures (23.1%), and head trauma (23.1%) were the most common indications for inappropriate scans [9]. Employing the same ACR guidelines, this Ethiopian study offers a relevant comparison to our findings, though it included both emergency and scheduled CT examinations. Our methodology, which involves evaluating request quality prior to appropriateness assessment, is supported by the authors’ emphasis on the critical need for thorough imaging requests to convey clinical justification. To standardize the imaging referrals quality evaluation, we used the RI-RADS categories [15, 16]. This system enables radiologists to evaluate the completeness of provided information and communicate this assessment in reports. By incorporating RI-RADS ratings, radiologists furnish feedback to requesting physicians, creating a “rating sheet” that promotes the provision of accurate and comprehensive clinical details [17]. Initially, we planned to assess appropriateness solely for requests rated adequate by RI-RADS (A or B). However, a shortage of adequate requests, primarily due to missing diagnostic questions, necessitated expanding our evaluation to include inadequate requests (RI-RADS C or D) where appropriateness could still be determined from available data. Evaluating inadequate requests for appropriateness introduced a potential bias: the data collectors’ assessments might have been skewed due to the absence of crucial clinical details, potentially masking diverse clinical presentations. For example, a headache request lacking “red flag” information could lead to inaccurate appropriateness judgments. On the other hand, the inclusion of inadequate requests proved important for the methodology, as these CT examinations were indeed performed and therefore offer insights into quality improvement.

To increase the level of quality and appropriateness of radiological requests, it is important on the one hand to integrate guidelines into clinical workflows and decision support systems within electronic health records, and on the other hand to implement robust mechanisms for the prospective justification of radiological examinations, ensuring that the rationale for each examination is clearly documented in the patient’s medical record. By requiring specific signs, symptoms, or diagnostic queries before submission, such a system would necessitate physicians completing a structured questionnaire with predefined answers (e.g., present/absent, duration). This would minimize radiologist misinterpretation and facilitate the retrieval of precise clinical data. To increase the level of appropriateness of head CT requests, one option would be to develop educational programs sharing the ACR criteria with the ED. Another option to increase confidence in appropriateness would require making the emergency team aware of the ESR iGuide as an accessible and trustworthy resource for requesting physicians.

Moreover, the use of artificial intelligence, particularly through the text analysis of large language models (LLMs) [31], presents a promising avenue for influencing imaging referral evaluations. Physicians could input their radiology requests into the hospital’s electronic system and receive immediate feedback on RI-RADS grading or ACR appropriateness, allowing for real-time adjustments to request completeness. The validity of using various LLMs as a tool for evaluating the quality and appropriateness of radiology exam requests across a range of clinical cases is a topic for future studies.

A careful interpretation of our study’s results is warranted, given several inherent limitations in addition to the previously mentioned potential bias related to the analysis of incomplete requests. It should be acknowledged that written radiology requests may not encompass the entirety of information shared by referring physicians, as supplementary details might be conveyed verbally. Nevertheless, complete written imaging requests are crucial to reduce the risk of errors and miscommunication. Due to its single-center design and the focus on head CT examination, the study’s conclusions may not be universally applicable. The exclusive inclusion of ED patients limits the applicability of our findings to other patient populations, such as those in outpatient or inpatient settings. Furthermore, the heterogeneous nature of our patient cohort complicates direct comparisons with other clinical contexts. Conversely, the consecutive enrollment of imaging referrals ensured a realistic representation of the head CT demands faced by radiologists in emergency clinical practice. Finally, the potential impact of various factors on request completeness and appropriateness was not investigated in this study. For instance, future studies could assess whether the level of appropriateness and quality of radiological requests correlates with the referring physician’s clinical experience, to implement targeted educational pathways.

## Conclusions

This research validates the prevalent practice of ED physicians requesting excessive head CTs, mostly with incomplete and often with inappropriate referrals, eventually leading to unnecessary radiation, environmental pollution, and increased healthcare costs. Improving the quality and appropriateness of radiological requests involves integrating guidelines into workflows and decision support, alongside implementing strong prospective justification processes that mandate clear documentation of the examination rationale in patient records. Additionally, improved healthcare staff training and a no-blame culture are recommended to reduce unnecessary imaging and defensive medicine.

## Data Availability

The data that support the findings of this study are available from the corresponding author, upon reasonable request.

## Abbreviations

ACR: American College of Radiology
CT: Computed tomography
ED: Emergency department
ESR: European Society of Radiology
LLM: Large language model
MRI: Magnetic resonance imaging
NPV: Negative predictive value
PPV: Positive predictive value
RI-RADS: Reason for exam imaging reporting and data system
RR: Relative risk
